# Detection of short tandem repeat expansions on a targeted neurological gene panel using STRipy improves the diagnostic rate for ataxias

**DOI:** 10.1093/braincomms/fcag092

**Published:** 2026-03-16

**Authors:** Carolin K Scriba, Chiara Folland, Michael Black, Jessica Baker, Daniel Abromeit, Samantha Saw, Mei-Ting Chiew, Rebecca Gooding, Nigel G Laing, Mark R Davis, Gianina Ravenscroft

**Affiliations:** Centre for Medical Research, University of Western Australia, Harry Perkins Institute of Medical Research, Perth, WA 6009, Australia; Department of Diagnostic Genomics, PathWest Laboratory Medicine, Nedlands, WA 6009, Australia; Centre for Medical Research, University of Western Australia, Harry Perkins Institute of Medical Research, Perth, WA 6009, Australia; Department of Diagnostic Genomics, PathWest Laboratory Medicine, Nedlands, WA 6009, Australia; Department of Diagnostic Genomics, PathWest Laboratory Medicine, Nedlands, WA 6009, Australia; Department of Diagnostic Genomics, PathWest Laboratory Medicine, Nedlands, WA 6009, Australia; Department of Diagnostic Genomics, PathWest Laboratory Medicine, Nedlands, WA 6009, Australia; Department of Diagnostic Genomics, PathWest Laboratory Medicine, Nedlands, WA 6009, Australia; Department of Diagnostic Genomics, PathWest Laboratory Medicine, Nedlands, WA 6009, Australia; Centre for Medical Research, University of Western Australia, Harry Perkins Institute of Medical Research, Perth, WA 6009, Australia; Department of Diagnostic Genomics, PathWest Laboratory Medicine, Nedlands, WA 6009, Australia; Centre for Medical Research, University of Western Australia, Harry Perkins Institute of Medical Research, Perth, WA 6009, Australia

**Keywords:** STRs, ataxia, diagnostic screening, neurogenetics

## Abstract

Short tandem repeat expansions are associated with over 50 diseases, many with primary neurological presentations. Despite the prevalence of short tandem repeat expansion disorders, genetically diagnosing these conditions is complicated by a lack of efficient and comprehensive diagnostic screening approaches. We integrated a new short tandem repeat genotyping tool, STRipy, into the analytical workflow for short-read sequencing data generated by the comprehensive neurological disease gene panel used in the Diagnostic Genomics Department, PathWest Laboratory Medicine. We tested STRipy on Versions 6 and 7 of the panel. Version 6 already included probes covering five short tandem repeat expansion loci in the following genes: *CACNA1A*, *PPP2R2B*, *TBP*, *NOP56* and *RFC1*. Additional probes targeting 13 neurological disease short tandem repeat expansion loci were designed and included in Version 7. All expansions detected by STRipy were validated and sized using PCR-based diagnostic techniques. Four hundred and eighteen patients with ataxia were tested on Version 6 of the panel, and 61 (14.6%) had reportable pathogenic variants, including 11 patients with pathogenic repeat expansions detected by STRipy. Sixty-seven ataxia patients were tested on Version 7 of the panel, and 15 (22.4%) had reportable pathogenic variants, including three repeat expansions detected by STRipy. Therefore, STRipy contributed 18.0% and 20.0% of the solved cases from Version 6 and 7 of the ataxia subpanels, respectively. STRipy accurately identified and sized loci with shorter pathogenic repeat thresholds where the expansion was smaller than the read length. In addition to increased diagnostic yield, implementation of STRipy into diagnostic analysis pipelines has streamlined clinical diagnosis of short tandem repeat expansion disorders.

## Teaser

Scriba *et al.* evaluated integration of the tool STRipy into the PathWest neurological gene panel for the detection of known short tandem repeat expansions. STRipy improved diagnostic yield in ataxia cases, contributing to 18–20% of solved cases. It reliably detected smaller expansions, streamlining diagnosis of repeat expansion disorders.

## Graphical Abstract

**Graphical Abstract:**
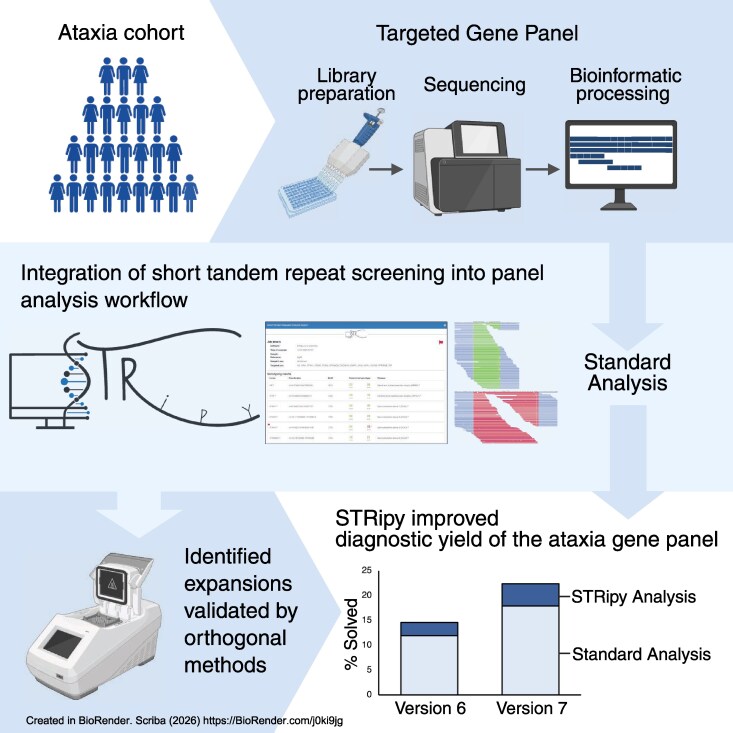

## Introduction

Neurogenetic disorders often exhibit heterogeneity within individual diseases while demonstrating high levels of clinical overlap between separate disease entities.^1,2^ Hundreds of genes have been associated with neurological conditions. For the past 10 years, short-read, massively parallel sequencing (MPS) technologies have enabled neurogenetic diagnostics to evolve from time-intensive single gene tests to high-throughput screens, testing hundreds of genes at once.^3,4^

Short tandem repeats (STRs) are short sequences of DNA between two and six nucleotides that are repeated consecutively within the genome. These repetitive regions display high rates of instability and are prone to expansion. More than 50 STR expansions have been associated with disease, the majority of which present as primary neurological conditions.^5^ Eighteen of these cause hereditary ataxias, accounting for the most common forms.^6^ Overall, STR expansion diseases are estimated to affect ∼1 in 3000 people.^7^ Despite their prevalence, the repetitive nature of these loci has made their detection challenging, and standard diagnostic practice continues to rely on single gene, PCR-based assays. Therefore, an adaptable high-throughput method for the screening of STR expansion disorders would provide great utility for neurogenetic diagnostics, improving both efficiency and diagnostic yield.

Several tools have been developed to identify STR expansions from short-read sequencing data.^8-13^ In 2022, the open-source software, STRipy, was released.^14^ STRipy provides a graphical user interface that can genotype loci from its catalogue of all known pathogenic STR expansions. The software uses ExpansionHunter as the backend genotyper and integrates the tool REviewer to provide simple visual presentation of the predicted genotype and detection of any phenotype altering interruptions. To enable automated, high-throughput analysis of STR expansion loci, Halman *et al*.^14^ also developed a pipeline version of STRipy.

We integrated the STRipy pipeline into the analytical workflow for data generated by the comprehensive MPS neurological disease gene panel used at PathWest’s Diagnostic Genomics department. The panel consists of more than 500 clinically relevant genes and is used to test approximately a thousand patients for neurogenetic conditions every year. Probes targeting additional STR expansion loci were incorporated into the newest version (Version 7) of the panel and 455 patients were screened for pathogenic expansions. We also conducted retrospective screening of ataxia patients who were tested on Version 6 of the panel (*n* = 418), as five STR expansion loci associated with ataxia phenotypes were already covered. Here, we demonstrate that STRipy is a valuable tool for screening known STR expansion disease loci from short-read targeted panel data, resulting in a substantially improved diagnostic yield for ataxias.

## Materials and methods

### Panel sequencing

The PathWest neurological disease targeted MPS gene panel (Version 6) consisted of 559 genes associated with numerous neurological disease phenotypes. Custom oligo design for target enrichment and panel manufacture were performed by TWIST Biosciences with library preparation, according to manufacturer’s instructions (EF workflow kit; Twist Bioscience, product nos. 101901 and 103904). Libraries were sequenced on the NextSeq 550/500 system using the Mid output cartridge (Illumina, Version 2.5, Cat no. SY-415-1002) with an average coverage of 300× across the panel and 50× for repeat regions. Raw sequencing data were processed using the DRAGEN enrichment pipeline, aligning to GRCh38. Analysis of sequencing data for single nucleotide variants (SNV) and small indels was performed using the Alissa Interpret Research software package (Agilent Technologies). Patients were analysed on *in silico* phenotypic subpanels corresponding to their clinical features. Copy number variants (CNVs) were detected as described by Beecroft *et al*..^3^

### Additional probes added to the PathWest neurological targeted gene panel

Four STR loci (SCA6, SCA12, SCA17 and SCA36) in genes associated with spinocerebellar ataxia (SCA) were already covered on Version 6 of the panel because non-repeat associated pathogenic variants have been reported within these genes. Probes for the *RFC1* locus were also already present as they were introduced to assess if manual inspection of binary alignment map (BAM) files could be used as a screening tool.^15^

To facilitate the detection of STR expansions from panel data, additional probes were ordered for Version 7 to cover loci for the following repeat expansion disorders: spinal and bulbar muscular atrophy (SBMA), dentatorubral-pallidoluysian atrophy (DRPLA), SCA1, SCA2, SCA3, SCA7, SCA8, SCA10, *C9Orf72* motor neuron disease/frontotemporal dementia (MND/FTD), SCA27B, Huntington disease-like 2 (HDL2), Huntington’s disease (HD) and neuronal intranuclear inclusion disease (NIID). Genes and oligo coordinates are displayed in Supplementary Table 1.

### Incorporating STRipy pipeline into the diagnostic workflow

**Figure 1:**
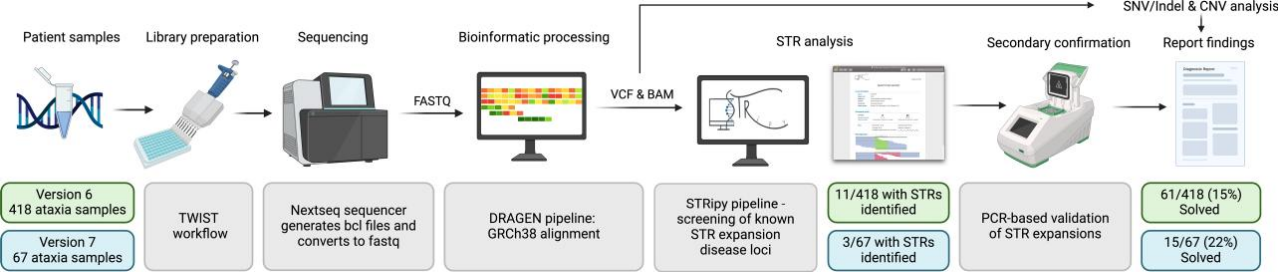
**Integration of STRipy into the PathWest diagnostic analysis workflow.** For Version 6 of the panel, STRipy was run retrospectively on existing sequencing data. This figure illustrates the updated diagnostic workflow used for Version 7 onwards. STRipy was integrated such that it runs in parallel with SNV/Indel and CNV analysis. The STRipy pipeline takes BAM files as input and generates user-friendly html reports. Abbreviations: VCF = variant call file; BAM = binary alignment map; STR = short tandem repeat; PCR = polymerase chain reaction; SNV = single nucleotide variant; CNV = copy number variant. Created in BioRender. Folland, C. (2025) https://BioRender.com/jk55i8g.

For automated, high-throughput analysis of STR expansion loci, we used the STRipy pipeline, a command-line version of STRipy (available at https://gitlab.com/andreassh/stripy-pipeline). Using a docker installation, the STRipy pipeline was integrated into the existing PathWest Diagnostic Genomics in-house bioinformatics analysis workflow (Fig. 1). The code and computational resources required are provided in the Supplementary Materials: Methods. The STR repeat loci that were analysed included all those associated with neurological conditions listed in the STRipy online database (https://stripy.org/database), including those covered in panel Versions 6 and 7 (see Supplementary Table 1). Loci that were not covered in Versions 6 and/or 7 were included in the analysis to account for STRipy capturing off-target expansions. A full list of regions analysable by STRipy using data from Version 6 and/or 7 of the panel is available in Supplementary Table 2. The pathogenic thresholds listed in the STRipy online database were used.^14^ A minimum of 20× coverage of each repeat region is required for STRipy to detect repeats.

**Figure 2:**
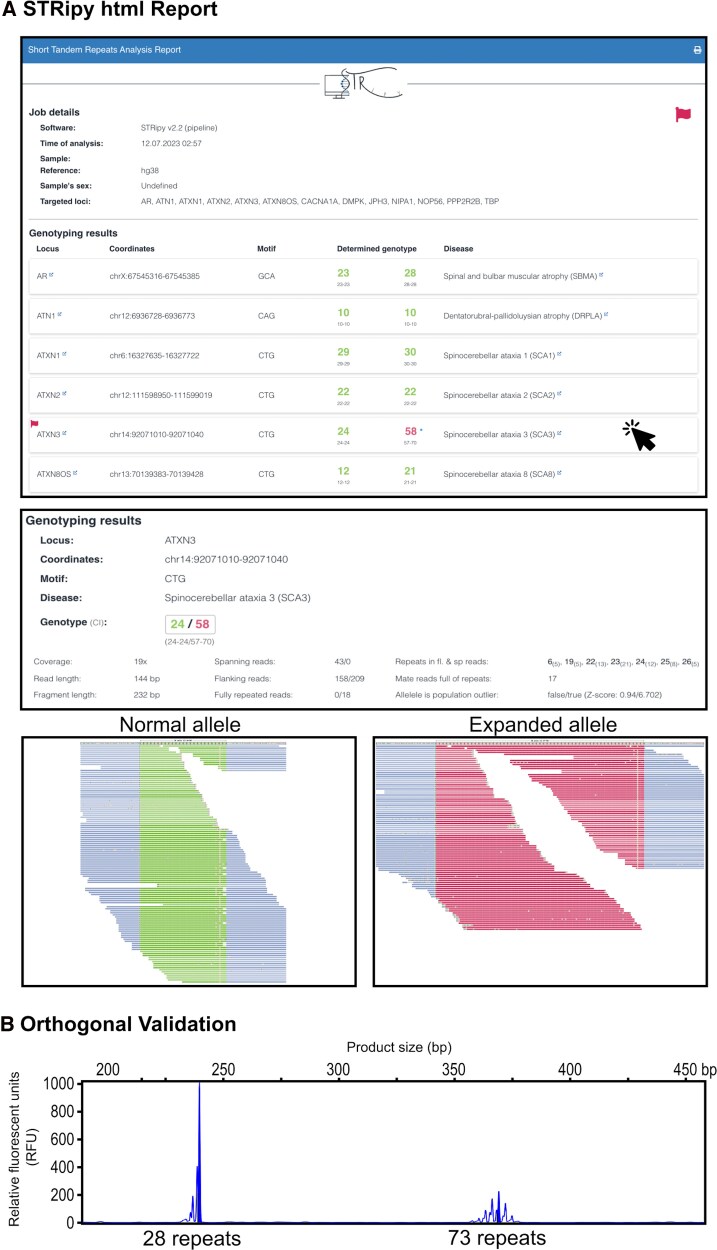
**Example of expansion detected by STRipy.** (**A**) Example html report generated by STRipy from panel data. This report shows a pathogenic expansion at the SCA3 locus. The html report summarizes genotyping results from all loci screened by STRipy. Any loci with expansions that pass the pathogenic threshold are labelled with a red flag icon that is easy to identify. The report is interactive; selecting a tab of interest produces a drop-down view including the genotype, coverage, read length and the number of spanning, flanking and fully repeated reads. There are also REViewer visualizations of the normal and expanded allele, which assist with interpretation of the result. (**B**) Orthogonal validation of the SCA3 repeat expansion using flanking PCR confirms presence of an expanded allele at 73 repeats.

Outputs consisted of html files summarizing results for each sample in an interactive report (Fig. 2) and a text file that indicated any intermediate or pathogenic results for all samples in a sequencing run. The html files show the gene, motif, coordinates, estimated number of repeats, threshold indicators, disease name and repeat alignment map for each STR locus analysed.

### Expansion controls

Available positive control samples with expansions that had been detected and sized through standard NATA-accredited diagnostic workflows were tested on Version 7 of the panel. The data were analysed by the STRipy pipeline to determine whether the software was able to accurately detect known pathogenic expansions.

### Running STRipy on panel data

**Version 6**: Sequencing data generated from all ataxia samples previously tested on Version 6 of the panel (*n* = 418, 197 XX, 222 XY) were retrospectively tested using the STRipy pipeline.

**Version 7**: Following the incorporation of additional probes to Version 7 of the panel, the STRipy pipeline was implemented for routine screening of sequencing data for all patients tested on the panel (*n* = 455, 220 XX, 235 XY), including 67 (36 XX, 31 XY) ataxia patients.

### Validation of STR expansions detected by STRipy

DNA samples from patients where STRipy indicated the presence of an expanded allele from the panel data were tested using the standard diagnostic workflow for repeat disorders to confirm the presence and size of the expanded allele. Individual techniques are detailed in the Supplementary Materials: Methods and Supplementary Table 3.

### Assessment of expansion in *C9orf72*

Due to the high prevalence of *C9orf72* expansions in MND/FTD patients, the diagnostic workflow first screens for the expansion, and patients testing negative for the expansion then cascade to gene panel testing. Eighty-two samples from clinically diagnosed MND/FTD patients negative for the *C9orf72* expansion were tested on Version 7 of the panel and analysed using flanking PCR to size alleles in the normal range. Samples indicating a single normal allele were tested by RP-PCR to determine whether the patient was homozygous for the normal sized allele or harboured an expansion (Supplementary Materials: Methods).

### Statistical analysis

Statistical comparison of STRipy output and validated allele sizes for the *C9orf72* repeat region was performed with Prism (GraphPad Software, Version 10.2.0(335)). The correlation between validated allele sizes at the *C9orf72* locus and STRipy predictions made with four or more spanning reads was assessed using Pearson’s product-moment correlation.

## Results

### Retrospective screening of the ataxia samples tested on Version 6 of the panel

Of the 418 patient samples tested on Version 6 of the panel, 12% (*n* = 50) had genetically resolved outcomes following standard analysis for indels, SNVs and CNVs. The cohort data was then run through the STRipy pipeline. STRipy indicated the presence of pathogenic expansions in 11 samples across multiple loci: four expansions associated with SCA6, three expansions associated with SCA36, and to our surprise two expansions associated with SCA1, and two expansions associated with SCA3 (Table 1). We did not expect to observe coverage of *ATXN1* and *ATXN3*, associated with SCA1 and SCA3, as probes for these loci were not included in Version 6 of the panel. Inspection of the REviewer outputs and BAM files at the SCA1 and SCA3 loci for these samples confirmed the presence of correctly mapped reads, with sequence matching that of the region flanking the repeat. Expansions in both SCA1 (*ATXN1*) and SCA3 (*ATXN3*) consist of CAG repeat motifs.^5,6^ This is also the repeat motif present in SCA6 (*CACNA1A*), SCA12 (*PPP2R2B*) and SCA17 (*TBP*),^5,6^ all of which are targeted in Version 6 of the panel. We hypothesize that non-specific binding of these probes captures the SCA1 and SCA3 loci, including them into the enriched library where they undergo clonal amplification and sequencing. This would provide enough coverage of these regions for STRipy to call the presence of an expansion at these loci.

**Table 1:** Pathogenic expansions detected on the Version 6 ataxia subpanel

		Locus details				STRipy output				Confirmed size	
Sample ID	Sex	Locus	Normal Range	Pathogenic threshold	Repeat	Allele 1 (repeats)	Spanning reads	Allele 2 (repeats)	Spanning reads	Allele 1 (repeats)	Allele 2 (repeats)
V6POS1	F	SCA1	6–32	≥39	CAG	30 (30–30)	35	50 (49–54)	0	30 (±1)	42 (±1)
V6POS2	M	SCA1	6–32	≥39	CAG	29 (29–29)	31	44 (44–44)	4	29 (±1)	44 (±1)
V6POS3	F	SCA3	12–44	≥56	CAG	24 (24–24)	43	58 (57–70)	0	28 (±1)^a^	73 (±2)^a^
V6POS4	F	SCA3	12–44	≥56	CAG	15 (15–15)	90	51 (51–60)	0	19 (±1)^a^	76 (±2)^a^
V6POS5	M	SCA36	3–14	≥25	GGCCTG	6 (6–6)	34	21 (19–23)	0	7 (±1)	>90
V6POS6	M	SCA36	3–14	≥25	GGCCTG	3 (3–3)	47	27 (27–31)	0	4 (±1)	>90
V6POS7	F	SCA36	3–14	≥25	GGCCTG	10 (10–10)	40	24 (24–27)	0	10 (±1)	>90
V6POS8	M	SCA6	4–18	≥21	CAG	13 (13-13)	44	22 (22–22)	43	13 (±1)	22 (±1)
V6POS9	F	SCA6	4–18	≥21	CAG	12 (12–12)	44	22 (22–22)	40	12 (±1)	22 (±1)
V6POS10	F	SCA6	4–18	≥21	CAG	13 (13–13)	43	22 (22–22)	30	13 (±1)	22 (±1)
V6POS11	F	SCA6	4–18	≥21	CAG	13 (13–13)	38	22 (22–22)	38	13 (±1)	22 (±1)

^a^ *ATXN3* (associated with SCA3) has a polymorphic region directly preceding the repeat locus where SNPs common in the population can result in additional repeats. Standard diagnostic practice includes this region for calculating the number of repeats from the PCR assay as the sequence is not detected by this technique. This results in four additional repeats calculated by the PCR assay compared to STRipy.

All expansions detected by STRipy, including for SCA1 and SCA3, were confirmed and accurately sized by orthogonal diagnostic methods. The concordance between allele sizes determined by STRipy and the confirmatory diagnostic assays is shown in Table 1.

The implementation of the STRipy pipeline for the few loci covered by Version 6 of the panel alone increased the diagnostic yield of the ataxia *in silico* panel from 12.0% to 14.6% overall (*n* = 61), with expansion detected by STRipy contributing 18.0% of the solved cases. Of note, STRipy detected a *NOP56* expansion in one patient exhibiting both ataxia and neuropathy with a strong autosomal dominant family history. The patient had previously been shown to harbour a *PMP22* duplication, following original panel data analysis, and therefore was unlikely to undergo further testing.

### Implementation of STRipy pipeline for Version 7 of the neurological gene panel

In Version 7 of the panel, we incorporated additional oligos to target repeat loci that were previously not covered on Version 6 (Supplementary Table 1). We first tested known positive controls that were available for the expansions associated with DRPLA, HD, SBMA, SCA2, SCA7, SCA8, SCA27B and the *C9orf72* repeat associated with MND/FTD. Excluding the *C9orf72* expansion, STRipy indicated the presence of an expansion for all positive control samples tested, with varying degrees of accuracy in relation to the repeat size (Table 2).

**Table 2:** Control samples tested on Version 7 of the neurological gene panel

		Locus details				STRipy output				Confirmed size	
Sample ID	Sex	Locus	Normal Range	Pathogenic threshold	Repeat	Allele 1 (repeats)	Spanning reads	Allele 2 (repeats)	Spanning reads	Allele 1 (repeats)	Allele 2 (repeats)
MNDCNTRL	F	MND/FTD	1–19	>24	GGGGCC	2 (2–2)	32	19 (16–23)	0	2 (±1)	>50
DRPLACNTRL	F	DRPLA	7–23	≥49	CAG	10 (10–10)	125	49 (49–56)	0	10 (±1)	68 (±1)
HDCTRL1	F	HD	9–26	≥36	CAG	20 (20–20)	78	40 (40–40)	8	20 (±1)	40 (±1)
HDCTRL2	M	HD	9–26	≥36	CAG	22 (22–22)	76	53 (53–65)	0	22 (±1)	98 (±1)
SBMACTRL	M	SBMA	17–35	≥40	CAG	42 (42–42)	12	NA	NA	41 (±1)	NA
SCA2CTRL	M	SCA2	13–31	≥35	CAG	22 (22–22)	87	37 (37–37)	19	22 (±1)	36 (±2)
SCA7CTRL	M	SCA7	4–35	≥36	CAG	13 (13–13)	25	49 (49–52)	0	13 (±1)	46 (±2)
SCA8CTRL	F	SCA8	2–37	≥80	CTG	59(43–66)	0	70 (58–83)	0	101 (±3)	154 (±3)

Since implementation of Version 7 of the panel, 455 samples have been screened, of which 67 samples were analysed on the *in silico* ataxia subpanel. The overall diagnostic yield for the ataxia panel was 22.4% (*n* = 15), including three samples with expansions detected by STRipy (SCA1, SCA2 and SCA3), confirmed and sized by accredited diagnostic testing (Table 3). Incorporating STRipy into the analysis workflow increased diagnostic yield for the ataxia subpanel from 17.9% to 22.4%, contributing 20% of solved cases.

**Table 3:** Pathogenic expansions detected on Version 7 of the neurological panel

		Locus details				STRipy output				Confirmed size	
Sample ID	Sex	Locus	Normal Range	Pathogenic threshold	Repeat	Allele 1 (repeats)	Spanning reads	Allele 2 (repeats)	Spanning reads	Allele 1 (repeats)	Allele 2 (repeats)
V7POS1	M	SCA1	6–32	≥39	CAG	30 (30–30)	53	48 (48–51)	0	31 (±1)	42 (±1)
V7POS2	F	SCA2	13–31	≥35	CAG	22 (22–22)	46	38 (38–38)	13	22 (±1)	38 (±2)
V7POS3	M	SCA3	12–44	≥56	CAG	18 (18–18)	148	51 (51–60)	0	22 (±1) a	77 (±1) a
V7POS4	M	HD	9–26	≥36	CAG	28 (28–28)	46	40 (40–40)	11	28 (±1)	40 (±1)

^a^ *ATXN3* (associated with SCA3) has a polymorphic region directly preceding the repeat locus where SNPs common in the population can result in additional repeats. Standard diagnostic practice includes this region for calculating the number of repeats from the PCR assay as the sequence is not detected by this technique. This results in four additional repeats calculated by the PCR assay compared to STRipy.

In addition to this, STRipy was able to detect the presence of, and accurately genotype, the CAG repeat expansion associated with HD in a sample that was tested on the dystonia/Parkinson’s disease subpanel (Table 3).

### Accuracy of STRipy calls and utility for screening

STRipy displayed greater accuracy for loci with shorter pathogenic repeat thresholds, where the size of the expansion was smaller than the read length. All pathogenic expansions called by STRipy with spanning reads were completely concordant with PCR-based sizing (Tables 1–3).

For some larger pathogenic expansions, STRipy could be used in a screening capacity to trigger further testing and accurate sizing. For example, STRipy could reliably size smaller alleles and indicate the presence of larger expansions associated with SCA1, SCA3 and SCA36, which could then be validated and sized using orthogonal methods.

For loci with a broader range of non-pathogenic allele sizes, such as *FGF14* and *RFC1*, the utility of STRipy is limited to the detection of smaller, normal alleles to exclude these loci as differential diagnoses, where indicated by the phenotype. The *RFC1* locus presents an added challenge for detecting pathogenic alleles as there are multiple repeat motifs associated with the locus. When alternate repeats are present, the coverage of the locus drops, likely due to reduced target enrichment as probes compliment the reference sequence. We have previously shown that manually assessing the BAM at this locus is useful for screening for the presence of biallelic expansions.^15^ STRipy improves the genotyping of normal alleles but is also limited by sequence coverage and read length for the assessment of expanded alleles. Though not comprehensive, this screening can reduce the number of samples that require testing by orthogonal methods.

Pathogenic expansions in *C9orf72* are the underlying cause of a significant proportion of MND/FTD, including up to 50% of familial cases and 10% of sporadic cases.^16^ The pathogenic threshold for *C9orf72* expansions is generally reported as >60 hexanucleotide repeats, though this threshold is not well defined, and expansions often reach thousands of repeats.^17^ REviewer visualizations for this locus indicated poor mapping quality within some samples analysed by STRipy, prompting further assessment.

Patients clinically diagnosed with MND/FTD are currently tested for the expansion by a PCR-based assay prior to panel testing. Eighty-two patients tested negative following flanking PCR and were consequently tested on Version 7 of the panel to identify alternate causes of disease. This enabled comparison of *C9orf72* non-pathogenic genotypes between STRipy and the PCR-based assay.

**Figure 3:**
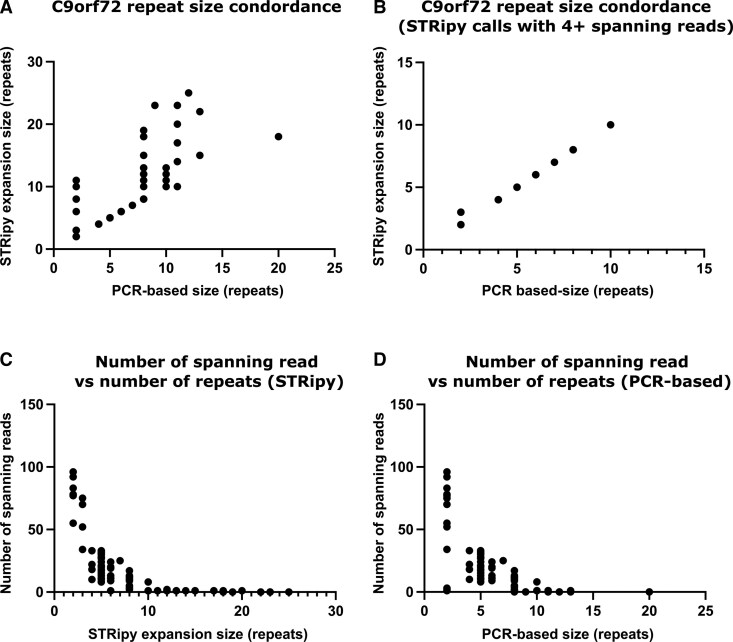
**C9orf72 repeat size concordance. All graphs relate to the *C9orf72* repeat locus associated with MND/FTD, each data point represents allele 2 from a patient tested for C9orf72 (*n* = 82).** (**A**) Displays the number of repeats predicted by STRipy compared to the validated allele size, using flanking PCR. (**B**) Shows the correlation between the number of repeats predicted by STRipy compared to the validated allele size using flanking PCR but only includes alleles where four or more spanning reads were present for the STRipy prediction (*n* = 50). Pearson’s correlation coefficient was 0.9925, *P* < 0.0001, indicating a strong correlation between STRipy genotype calls and PCR-based sizing. (**C**) Displays the number of spanning reads available and the number of repeats predicted by STRipy for each allele. (**D**) Shows the number of spanning reads utilized for the STRipy prediction compared to the validated allele size using flanking PCR.

There was substantial discordance between predicted STRipy genotypes and PCR-based assessment of *C9orf72* in several samples, making interpretation of STRipy calls for this locus challenging. The *C9orf72* locus shows greater variability in the number of spanning reads for smaller alleles than expected (Fig. 3). This was reflected in the discordant results seen for some samples with smaller alleles (Fig. 3) and may indicate inconsistencies with sequencing or read mapping in this GC-rich region. Alleles with four or more spanning reads at the *C9orf72* locus (*n* = 50) showed a high level of correlation (r = 0.9925, *P* < 0.0001) between STRipy genotype calls and PCR-based sizing (Fig. 3).

## Discussion

STR expansions are a common cause of multiple inherited neurological disorders.^7^ Presently, molecular diagnostics for STR expansions involve individual, repeat specific assays. Testing for these disorders therefore relies heavily on clinical assessment, which is often confounded by the phenotypic heterogeneity and clinical overlap between disorders.^5^ This often results in iterative testing, prolonging patient uncertainty. Due to lack of feasibility, diagnostic testing for many of the repeat loci is not routinely available at diagnostic laboratories in Australia. This requires send-away of samples and screening by an international service provider, often with considerable costs and lengthy time delays.

Here we used STRipy to screen short-read panel data for pathogenic expansions in a diagnostic setting. We demonstrate that integration of STRipy into panel analysis provides a high-throughput method for screening multiple STR expansion loci concurrently and significantly improves diagnostic yield for ataxia patients. Expansions were identified in 2.6% and 4.5% of all patients tested on Versions 6 and 7 of the ataxia subpanel, improving the diagnostic yield of these panels by 18% and 20%, respectively. This is comparable to other studies that have used ExpansionHunter to analyse exome sequencing data, identifying STR expansions in 0.24–4.4% of cases.^18-20^ We were also able to identify a pathogenic expansion associated with HD in a patient tested on the dystonia/Parkinson’s subpanel. The HD phenotype has considerable heterogeneity,^21,22^ which can lead to a delay in diagnosis for patients seeking informed family planning and clinical management. Our results show that detection of STR expansions using panel data is comparable to that of exome data and demonstrate that integrating STR calling into diagnostic analysis pipelines can increase diagnostic yield and improve testing efficiency.

Both read length and locus coverage impact detection of STR expansions from short-read sequencing.^18,23^ The primary advantages of targeted gene panels are their adaptability and the depth of coverage afforded. The high read depth provides greater confidence in the calls made. STRipy performed best for loci with lower pathogenic thresholds, showing complete concordance with orthogonal techniques where the size of the expanded allele was smaller than the read length.

STRipy was also able to successfully identify expansions in *ATXN1*, *ATXN3* and *NOP56* that exceeded the read length. Though unable to size larger expansions, the ability to accurately genotype smaller alleles provides a useful screening tool for expansions that have a narrow range for the normal allele present within the population. For example, pathogenic expansions in the *NOP56* gene associated with SCA36 generally exceed 1000 hexanucleotide repeats (∼6 kb); however, the reference range within population controls is between 3 and 14 repeats.^24,25^ In all *NOP56* cases detected by STRipy above this range, no spanning reads were present for the larger allele, indicating that the expansions were likely larger than predicted, and this was confirmed by orthogonal methods. As such, alleles indicated by STRipy to harbour more repeats than the upper bound of the reference range, particularly where no spanning reads are mapped, warrant further testing. For large expansions where the normal range is broader and may exceed read length, such as *FGF14*, *C9orf72* and *RFC1*, the utility of STRipy is limited to excluding the presence of an expansion where two normal alleles are detected with adequate spanning reads.

The capabilities of any analysis software are limited by the type and quality of data input. The ability to size larger expansions from the targeted panel data was reduced when compared to genome-wide sequencing data.^14^ From the alignment file, STRipy extracts all reads that overlap the STR locus and a 2 kb region flanking the repeat.^14^ Next, reads directly flanking the STR region, as well as fully repeated reads that have a pair in the extracted region, are included in the analysis file.^14^ The probes used here to capture regions of interest for the panel target regions of ∼200 bp. Targeting larger regions encompassing the repeat may improve capacity to resolve larger expansions from panel data by generating more reads with in-repeat read mates. This may also produce more accurate genotypes for the *C9orf72* locus.

The phenotypic heterogeneity observed across numerous STR expansion disorders complicates differential diagnosis and individual test selection. Integrating STR calling into short-read data analysis provides a comprehensive first-line screen. The testing captures a broader range of patient phenotypes, including patients with fewer differential features indicative of specific STR expansions who may not have otherwise been tested. One expansion that is likely to have been missed was discovered through the retrospective screening of ataxia patients tested on Version 6 of the panel. This patient had a pathogenic expansion in *NOP56*, in addition to a reported *PMP22* duplication associated with Charcot–Marie–Tooth (CMT) neuropathy. Clinical notes for the patient indicated both neuropathic and ataxic phenotypes. However, without the additional screening, all clinical features would have been attributed to the *PMP22* duplication, hindering informative familial testing. Dual diagnoses are often difficult to determine due to a blended phenotype. Despite this they have been reported to contribute up to 7% of diagnoses in patients tested using exome sequencing.^26-28^ As genomic testing becomes more comprehensive, identification of dual diagnoses may increase, improving the ability to delineate blended phenotypes.

Integration of STR screening tools into short-read MPS workflows also improves accessibility to STR expansion testing. Accredited diagnostic testing for *SCA36* expansions in *NOP56* is not currently available in Australia and many other countries.^29^ Prior to implementing STRipy into the sequencing analysis pipeline, our laboratory did not offer testing for SCA36. STRipy detected pathogenic *NOP56* expansions in three ataxia patients tested on Version 6 of the panel, contributing ∼5% of all genetically resolved ataxia cases. MPS gene panels are the first-line test for neurological disorders in many diagnostic laboratories. Since their widespread adoption, significant advances have been made in the analysis of short-read MPS data. Incorporation of additional tools is important to fully utilize data already generated. These tools provide substantial value without significant cost. Implementing STR detection software into diagnostic analysis pipelines facilitates screening for SCA36 and other rare STR expansions where routine diagnostic testing may not be readily available. Command-line implementation of STRipy enabled concurrent genotyping of all repeat loci for every sample tested. Individual reports were produced, which display all genotypes and alignment visualizations in a user-friendly and interactive display. This enabled high-throughput screening of STR expansions with minimal additional training or workload for analysts.

## Conclusion

Here, we demonstrate that screening pathogenic STR expansions using STRipy on a neurological gene panel accurately detects expansions and improves the diagnostic success rate for ataxias in a clinical diagnostic laboratory. STRipy offers a simple computational approach that comprehensively screens many loci in a single workflow, improving testing efficiency and reducing the cycles of iterative testing. This is particularly beneficial for diagnosing atypical phenotypes, identifying dual diagnoses, and for rare disorders where testing may not be routinely available. Preliminary work has also demonstrated the diagnostic utility of STRipy for diagnosing muscle-related repeat expansions from panel data, including identifying patients with pathogenic expansions in *DMPK* and *CNBP*, associated with myotonic dystrophy types 1 and 2 (DM1 and DM2).

## Supplementary Material

fcag092_Supplementary_Data

## Data Availability

The data that support the findings of this study are available upon request from the corresponding author.
