# Supplementary material for: Detection of short tandem repeat expansions on a targeted neurological gene panel using STRipy improves the diagnostic rate for ataxias
Source: Brain Commun. 2026 Mar 16;8(2):fcag092. doi: 10.1093/braincomms/fcag092 (PMC13107179; doi:10.1093/braincomms/fcag092)
Supplement: fcag092_Supplementary_Data [file fcag092_supplementary_data.pdf]

SUPPLEMENTARY MATERIALS

PCR based diagnostic assays for validation of expansion detected by STRipy

SCA1, SCA2, SCA3 and SCA6

Flanking PCR was performed on all samples with suspected SCA1, SCA2, SCA3, and SCA6 expansions. Primer sequences, reaction components, and thermocycling conditions were adapted from Orr *et al.*, Kawaguchi *et al.*, Zhuckenko *et al.* and David *et al.*[1-4], and are provided in Supplementary Table 3. Following amplification, 1 µl of PCR product was combined with 11.875 µl of Hi-Di Formamide (Applied Biosystems Cat no. 4311320) and 0.125 µl of GeneScan LIZ600 size standard (Applied Biosystems Cat no. 4408399). To facilitate accurate sizing, samples underwent capillary electrophoresis fragment length separation performed on the ABI 3730 automated DNA Analyser. Allele fragments were sized using the software GeneMapper (Version 4.1) and the number of repeats were calculated from the fragment size.

SCA36

Flanking PCR and repeat-primed PCR was performed on three samples where STRipy indicated the presence of an expansion. Primer sequences, reaction components, and thermocycling conditions are provided in Supplementary Table 3. Following amplification, 1 µl of PCR product was combined with 11.875 µl of Hi-Di Formamide (Applied Biosystems Cat no. 4311320) and 0.125 µl of GeneScan LIZ600 size standard (Applied Biosystems Cat no. 4408399). Capillary electrophoresis fragment length separation was performed on the ABI 3730 automated DNA Analyser and the data analysed using GeneMapper software (Version 4.1).

HD

Flanking PCR was used to confirm allele sizes for patients with predicted HD expansions. Primer sequences, reaction components, and thermocycling conditions are provided in

Supplementary Table 3. Following amplification, 1 μl of PCR product was combined with 9 μl of Hi-Di Formamide (Applied Biosystems Cat no. 4311320) and 0.3 μl of GeneScan LIZ500 size standard (Applied Biosystems Cat no. 4322682). Capillary electrophoresis fragment length separation was performed on the ABI 3730 automated DNA Analyser. The data was analysed using GeneMapper software (Version 4.1) and the number of repeats were calculated from the fragment size.

C9orf72 PCR assays

The primer sequences, reaction components, and thermocycling conditions were adapted from Dejesus-Hernandez *et al.* and Warner *et al.*[5,6] and are provided in Supplementary Table 3. Following amplification, 1 μl of PCR product was combined with 11.875 μl of Hi-Di Formamide (Applied Biosystems Cat no. 4311320) and 0.125 μl of GeneScan LIZ600 size standard (Applied Biosystems Cat no. 4408399). The ABI 3730 automated DNA Analyser was used to perform capillary electrophoresis fragment length separation. The data was analysed using GeneMapper software (Version 4.1) and the number of repeats were calculated from the fragment size.

REFERENCES

1. Orr, H.T. *et al.* Expansion of an unstable trinucleotide CAG repeat in spinocerebellar ataxia type 1. *Nat Genet* **4**, 221-6 (1993).
2. Kawaguchi, Y. *et al.* CAG expansions in a novel gene for Machado-Joseph disease at chromosome 14q32.1. *Nat Genet* **8**, 221-8 (1994).
3. Zhuchenko, O. *et al.* Autosomal dominant cerebellar ataxia (SCA6) associated with small polyglutamine expansions in the alpha 1A-voltage-dependent calcium channel. *Nat Genet* **15**, 62-9 (1997).
4. David, G. *et al.* Cloning of the SCA7 gene reveals a highly unstable CAG repeat expansion. *Nat Genet* **17**, 65-70 (1997).
5. DeJesus-Hernandez, M. *et al.* Expanded GGGGCC hexanucleotide repeat in noncoding region of C9ORF72 causes chromosome 9p-linked FTD and ALS. *Neuron* **72**, 245-56 (2011).
6. Warner, J.P. *et al.* A general method for the detection of large CAG repeat expansions by fluorescent PCR. *J Med Genet* **33**, 1022-6 (1996).

Supplementary Table 3 | Details of PCR protocols used for confirmation of expansions detected by STRipy.

<table>
<tr><th>Assay</th><th>Primer Sequence</th><th>Reaction Components</th><th>Thermocycling Conditions</th></tr>
<tr><td rowspan="2">SCA1</td><td>F – 6FAM-
GCCAGACGCCGGGACAC
AAGG</td><td rowspan="2">20 µl reaction
2 µl HotStarTaq 10X PCR Buffer (15mM MgCl2)
1.6 µl dNTPs (5 mM)
0.5 µl FAM forward primer (50ng/µl)
0.5 µl Reverse primer (50ng/µl)
4 µl 5X Q solution
0.1µl HotStarTaq DNA Polymerase
5µl Target DNA (10ng/µl)
Nuclease free water to 20 µl</td><td rowspan="6"><table>
<tr><th>Cycles</th><th>Temperature</th><th>Time</th></tr>
<tr><td>1</td><td>95°C</td><td>15 min</td></tr>
<tr><td rowspan="3">15</td><td>94°C</td><td>45 sec</td></tr>
<tr><td>68°C
-0.5°C per cycle</td><td>1 min</td></tr>
<tr><td>72°C</td><td>1 min</td></tr>
<tr><td rowspan="3">20</td><td>94°C</td><td>45 sec</td></tr>
<tr><td>60°C
-0.5°C per cycle</td><td>45 sec</td></tr>
<tr><td>72°C</td><td>1 min</td></tr>
<tr><td>1</td><td>72°C</td><td>10 min</td></tr>
<tr><td>1</td><td>4°C</td><td>Hold</td></tr>
</table></td></tr>
<tr><td>R –
GGTGATGAGCCCCGGAG
CCCT</td></tr>
<tr><td rowspan="2">SCA2</td><td>F – 6FAM-
GGGCCCCTCACCATGTCG</td><td rowspan="2">20 µl reaction
2 µl HotStarTaq 10X PCR Buffer (15mM MgCl2)
1.6 µl dNTPs (5 mM)
1 µl FAM forward primer (50ng/µl)
1 µl Reverse primer (50ng/µl)
2 µl DMSO
0.1µl HotStarTaq DNA Polymerase
5µl Target DNA (10ng/µl)
Nuclease free water to 20 µl</td></tr>
<tr><td>R–
CGGGCTTGCGGACATTGG</td></tr>
<tr><td rowspan="2">SCA6</td><td>F – 6FAM-
CACGTGTCCTATTCCCCTG
TGATCC</td><td rowspan="2">20 µl reaction
2 µl HotStarTaq 10X PCR Buffer (15mM MgCl2)
1.6 µl dNTPs (5 mM)
1 µl FAM forward primer (50ng/µl)
1 µl Reverse primer (50ng/µl)
1.2 µl MgCl2 (25mM)
4 µl 5X Q solution
0.1µl HotStarTaq DNA Polymerase
5µl Target DNA (10ng/µl)
Nuclease free water to 20 µl</td></tr>
<tr><td>R –
TGGGTACCTCCGAGGGC
CGCTGGTG</td></tr>
</table>

<table>
<tr>
<td>SCA3</td>
<td>F – 6FAM-CCAGTGACTACTTTGATTCG
R – TGGCCTTTCACATGGATGTGAA</td>
<td>20 µl reaction
2 µl HotStarTaq 10X PCR Buffer (15mM MgCl2)
1.6 µl dNTPs (5 mM)
1 µl FAM forward primer (50ng/µl)
1 µl Reverse primer (50ng/µl)
4 µl 5X Q solution
0.1µl HotStarTaq DNA Polymerase
5µl Target DNA (10ng/µl)
Nuclease free water to 20 µl</td>
<td>
<table>
<tr><th>Cycles</th><th>Temperature</th><th>Time</th></tr>
<tr><td>1</td><td>95°C</td><td>15 min</td></tr>
<tr><td rowspan="3">15</td><td>94°C</td><td>45 sec</td></tr>
<tr><td>63°C
-0.5°C per cycle</td><td>1 min</td></tr>
<tr><td>72°C</td><td>1 min</td></tr>
<tr><td rowspan="3">20</td><td>94°C</td><td>45 sec</td></tr>
<tr><td>55°C
-0.5°C per cycle</td><td>45 sec</td></tr>
<tr><td>72°C</td><td>1 min</td></tr>
<tr><td>1</td><td>72°C</td><td>10 min</td></tr>
<tr><td>1</td><td>4°C</td><td>Hold</td></tr>
</table>
</td>
</tr>
<tr>
<td>SCA36 Flanking PCR</td>
<td>F- 6FAM-TTTCGGCCTGCGTTCGGG
R-AACGCAACCTCAGCGTCT</td>
<td>25 µl reaction
3.2 µl HotStarTaq 10X PCR Buffer (15mM MgCl2)
2.6 µl dNTPs (5 mM)
1.3 µl 7-deaza-dGTP (5mM)
1 µl FAM forward primer (50ng/µl)
1 µl Reverse primer (50ng/µl)
2.4 µl DMSO
0.2 µl HotStarTaq DNA Polymerase
5µl Target DNA (10ng/µl)
Nuclease free water to 25 µl</td>
<td>
<table>
<tr><th>Cycles</th><th>Temperature</th><th>Time</th></tr>
<tr><td>1</td><td>95°C</td><td>15 min</td></tr>
<tr><td rowspan="3">40</td><td>94°C</td><td>45 sec</td></tr>
<tr><td>60°C</td><td>45 sec</td></tr>
<tr><td>72°C</td><td>1 min</td></tr>
<tr><td>1</td><td>72°C</td><td>20 min</td></tr>
<tr><td>1</td><td>4°C</td><td>Hold</td></tr>
</table>
</td>
</tr>
<tr>
<td>SCA36 RP-PCR</td>
<td>P1- 6FAM-TTTCGGCCTGCGTTCGGG
P4-TACGCATCCCAGTTTGAGACGCAGGCCCAGGCCCAGGCCCAGGCC</td>
<td>20 µl reaction
2 µl HotStarTaq 10X PCR Buffer (15mM MgCl2)
1.6 µl dNTPs (10 mM with complete replacement of dGTP with 7-deaza-dGTP)
1 µl P1 primer (10uM)
0.5 µl P4 primer (10uM)</td>
<td>
<table>
<tr><th>Cycles</th><th>Temperature</th><th>Time</th></tr>
<tr><td>1</td><td>95°C</td><td>15 min</td></tr>
<tr><td>48</td><td>95°C
ramp 2.5°C per sec</td><td>30 sec</td></tr>
</table>
</td>
</tr>
</table>

<table>
<tr>
<td></td>
<td>P3-
TACGCATCCCAGTTTGAGA
CG</td>
<td>1 µl P3 primer (10uM)
1 µl DMSO
4 µl 5X Q solution
0.2 µl HotStarTaq DNA Polymerase
5µl Target DNA (10ng/µl)
Nuclease free water to 20 µl</td>
<td>
<table>
<tr><td></td><td>70°C
-0.3°C per cycle,
ramp 1.5°C per sec</td><td>30 sec</td></tr>
<tr><td></td><td>72°C
ramp 2.5°C per sec</td><td>3 min</td></tr>
<tr><td rowspan="3">20</td><td>95°C
ramp 2.5°C per sec</td><td>30 sec</td></tr>
<tr><td>58°C
ramp 1.5°C per sec</td><td>30 sec</td></tr>
<tr><td>72°C
ramp 2.5°C per sec</td><td>3 min</td></tr>
<tr><td>1</td><td>72°C</td><td>7 min</td></tr>
<tr><td>1</td><td>4°C</td><td>Hold</td></tr>
</table>
</td>
</tr>
<tr>
<td>HD</td>
<td>F - 6FAM-
ATGAAGGCCTTCGAGTCC
CTCAAGTCCTT

R -
GGCGGTGGCGGCTGTTG
CTGCTGCTGCTGC</td>
<td>25 µl reaction
2.5 µl X10 HotStarTaq 10X PCR Buffer (15mM MgCl2)
1.5 µl Magnesium Chloride (25mM)
0.5 µl 25 mM dNTPs
1 µl Forward primer (10mM))
1 µl Reverse primer (10mM))
5 µl Q solution
0.25 µl HotStarTaq DNA Polymerase
10µl Target DNA (10ng/µl)
Nuclease free water to 25 µl</td>
<td>
<table>
<tr><th>Cycles</th><th>Temperature</th><th>Time</th></tr>
<tr><td>1</td><td>95°C</td><td>15 min</td></tr>
<tr><td rowspan="3">30</td><td>98°C</td><td>45 sec</td></tr>
<tr><td>63°C</td><td>1 min,
30 sec</td></tr>
<tr><td>72°C</td><td>1 min</td></tr>
<tr><td>1</td><td>4°C</td><td>Hold</td></tr>
</table>
</td>
</tr>
<tr>
<td>C9ORF72 Flanking PCR</td>
<td>F-6FAM-
CAAGGAGGGAAACAACC
GCAGCC</td>
<td>20 µl reaction
2 µl HotStarTaq 10X PCR Buffer (15mM MgCl2)</td>
<td>
<table>
<tr><th>Cycles</th><th>Temperature</th><th>Time</th></tr>
</table>
</td>
</tr>
</table>

<table>
<tr>
<td></td>
<td>R-
GCAGGCACCGCAACCGCAG</td>
<td>1.6 µl dNTPs (10 mM with complete replacement of dGTP with 7-deaza-dGTP)
0.5 µl FAM forward primer (10 pmol/µl)
0.5 µl Reverse primer (10 pmol/µl)
1 µl DMSO
4 µl 5X Q solution
0.2 µl HotStarTaq DNA Polymerase
5µl Target DNA (10ng/µl)
Nuclease free water to 20 µl</td>
<td><table>
<tr><td>1</td><td>95°C</td><td>15 min</td></tr>
<tr><td rowspan="3">35</td><td>94°C</td><td>45 sec</td></tr>
<tr><td>60°C</td><td>45 sec</td></tr>
<tr><td>72°C</td><td>1 min</td></tr>
<tr><td>1</td><td>72°C</td><td>20 min</td></tr>
<tr><td>1</td><td>4°C</td><td>Hold</td></tr>
</table></td>
</tr>
<tr>
<td>C9ORF72 RP-PCR</td>
<td>P1-6FAM-
CAAGGAGGGAAACAACCGCAGCC<hr>P4-
TACGCATCCCAGTTTGAGACGGGCCCCGGCCCCGGCCCC<hr>P3-
TACGCATCCCAGTTTGAGACG</td>
<td>20 µl reaction
2 µl HotStarTaq 10X PCR Buffer (15mM MgCl2)
1.6 µl dNTPs (10 mM with complete replacement of dGTP with 7-deaza-dGTP)
1 µl P1 primer (10 pmol/µl)
0.5 µl P4 primer (10 pmol/µl)
1 µl P3 primer (10 pmol/µl)
1 µl DMSO
4 µl 5X Q solution
0.2 µl HotStarTaq DNA Polymerase
5µl Target DNA (10ng/µl)
Nuclease free water to 20 µl</td>
<td><table>
<tr><th>Cycles</th><th>Temperature</th><th>Time</th></tr>
<tr><td>1</td><td>95°C</td><td>15 min</td></tr>
<tr><td rowspan="3">48</td><td>95°C
ramp 2.5°C per sec</td><td>30 sec</td></tr>
<tr><td>70°C
-0.3°C per cycle,
ramp 1.5°C per sec</td><td>30 sec</td></tr>
<tr><td>72°C
ramp 2.5°C per sec</td><td>3 min</td></tr>
<tr><td rowspan="3">20</td><td>95°C
ramp 2.5°C per sec</td><td>30 sec</td></tr>
<tr><td>58°C
ramp 1.5°C per sec</td><td>30 sec</td></tr>
<tr><td>72°C
ramp 2.5°C per sec</td><td>3 min</td></tr>
<tr><td>1</td><td>72°C</td><td>7 min</td></tr>
<tr><td>1</td><td>4°C</td><td>Hold</td></tr>
</table></td>
</tr>
</table>

Reagent specifications:

- HotStarTaq DNA Polymerase - HotStarTaq DNA polymerase kit (QIAGEN Cat no. 203205)
- HotStarTaq 10X PCR Buffer- HotStarTaq DNA polymerase kit (QIAGEN Cat no. 203205)
- 5X Q solution - HotStarTaq DNA polymerase kit (QIAGEN Cat no. 203205)
- Magnesium Chloride (25mM) - HotStarTaq DNA polymerase kit (QIAGEN Cat no. 203205)
- dNTP mix used for HTT flanking PCR - 100mM of each (Promega Cat No. U1240)
- dNTP mix with complete replacement of dGTP - dATP (ThermoFisher Scientific Cat no. 18252-015), dTTP (ThermoFisher Scientific Cat no. 18255-018), dCTP (ThermoFisher Scientific Cat no. 18253-013) and 7-deaza-dGTP (Roche, Cat no 10988537001).
- dNTP mix used for all other assays – (Applied Biosystems Cat no. 362275)

Supplementary Table 1 | Expansion loci with probes included in Versions 6 and/or 7 of the neurological gene panel.

Regions covered in version 7

Gene	Disease	Disease	Motif	Normal range	Pathogenic Threshold	Region	Expansion co-ordinates	Oligo co-ordinates
AR	SBMA	Spinal and bulbar muscular atrophy (SBMA)	CAG	17-35	≥40	Coding	chrX:67545316-67545385	Chr X: 67,545,250-67,545,450
ATN1	DRPLA	Dentatorubral-pallidoluysian atrophy (DRPLA)	CAG	7-23	≥49	Coding	chr12:6936728-6936773	Chr 12: 6,936,650-6,936,850
ATXN1	SCA1	Spinocerebellar ataxia 1 (SCA1)	CAG	6-32	≥39	Coding	chr6:16327635-16327722	Chr 6: 16,327,600-16,327,800
ATXN2	SCA2	Spinocerebellar ataxia 2 (SCA2)	CAG	13-31	≥35	Coding	chr12:111598950-111599019	Chr 12: 111,598,900-111,599,100
ATXN3	SCA3	Spinocerebellar ataxia 3 (SCA3)	CAG	12-44	≥56	Coding	chr14:92071010-92071040	Chr 14: 92,070,900-92,071,100
ATXN7	SCA7	Spinocerebellar ataxia 7 (SCA7)	CAG	4-35	≥36	Coding	chr3:63912685-63912715	Chr 3: 63,912,600-63,912,800
ATXN8OS	SCA8	Spinocerebellar ataxia 8 (SCA8)	CTG	2-37	≥80	3′ UTR	chr13:70139383-70139428	Chr13: 70,139,300-70,139,500
ATXN10	SCA10	Spinocerebellar ataxia 10 (SCA10)	ATTCT	10-29	≥800	Intron	chr22:45795354-45795424	Chr 22: 45,795,250-45,795,550
C9ORF72	MND/FTD	Motor neuron disease and/or frontotemporal dementia (MND/FTD)	GGGGCC	1-19	≥24	Intron	chr9:27573528-27573546	Chr9: 27,573,450-27,573,650
FGF14	EA	Spinocerebellar ataxia 27B (SCA27B)	GAA	9-249	≥300	Intron	chr13:102161567-102161726	Chr13:102,161,500-102,161,800
JPH3	HD-like 2	Huntington disease-like 2 (HDL2)	CTG	6-27	≥41	3′ UTR	chr16:87604287-87604329	Chr 16: 87,604,200-87,604,400
HTT	HD	Huntington's disease (HD)	CAG	9-26	≥36	Coding	chr4:3074876-3074933	Chr 4: 3,074,800-3,075,000
NOTCH2NLC	NIID	Neuronal intranuclear inclusion disease (NIID)	GGC	7-39	≥90	5′ UTR	chr1:149390802-149390829	Chr 1: 149,390,700-149,390,900

Regions with coverage in version 6 and 7

Gene	Disease	Disease	Motif	Normal range	Pathogenic Threshold	Region	Expansion co-ordinates
CACNA1A	SCA6	Spinocerebellar ataxia 6 (SCA6)	CAG	4-18	≥21	Coding	chr19:13207858-13207897
NOP56	SCA36	Spinocerebellar ataxia 36 (SCA36)	GGCCTG	3-14	≥25	Intron	chr20:2652733-2652757
PPP2R2B	SCA12	Spinocerebellar ataxia 12 (SCA12)	CAG	7-31	≥51	5′ UTR	chr5:146878728-146878758
TBP	SCA17	Spinocerebellar ataxia 17 (SCA17)	CAG	25-42	≥43	Coding	chr6:170561907-170562015
RFC1	CANVAS	Cerebellar ataxia, neuropathy, and vestibular areflexia syndrome (CANVAS)	AAGGG, ACAGG, AAAAG	11-200 (AAAAG)	≥400 (AAGGG, ACAGG)	Intron	chr4:39348424-39348485

Supplementary Table 2 | Regions detected by STRipy. Expansion loci included in STRipy analysis of panel data. Loci that were not covered in Versions 6 and/or 7 (Probes included = No) were included in the analysis to account for STRipy capturing off-target expansions.

Gene	Disease	Disease	Motif	Normal range	Pathogenic Threshold	Region	Expansion co-ordinates	Probes included	Analysed by STRipy
AR	SBMA	Spinal and bulbar muscular atrophy (SBMA)	CAG	17-35	≥40	Coding	chrX:67545316-67545385	V7 only	V6 & V7
ATN1	DRPLA	Dentatorubral-pallidoluysian atrophy (DRPLA)	CAG	7-23	≥49	Coding	chr12:6936728-6936773	V7 only	V6 & V7
ATXN1	SCA1	Spinocerebellar ataxia 1 (SCA1)	CAG	6-32	≥39	Coding	chr6:16327635-16327722	V7 only	V6 & V7
ATXN10	SCA10	Spinocerebellar ataxia 10 (SCA10)	ATTCT	10-29	≥800	Intron	chr22:45795354-45795424	V7 only	V6 & V7
ATXN2	SCA2	Spinocerebellar ataxia 2 (SCA2)	CAG	13-31	≥35	Coding	chr12:111598950-111599019	V7 only	V6 & V7
ATXN3	SCA3	Spinocerebellar ataxia 3 (SCA3)	CAG	12-44	≥56	Coding	chr14:92071010-92071040	V7 only	V6 & V7
ATXN7	SCA7	Spinocerebellar ataxia 7 (SCA7)	CAG	4-35	≥36	Coding	chr3:63912685-63912715	V7 only	V7 only
ATXN8OS	SCA8	Spinocerebellar ataxia 8 (SCA8)	CTG	2-37	≥80	3′ UTR	chr13:70139383-70139428	V7 only	V6 & V7
C9ORF72	MND/FTD	Motor neuron disease and/or frontotemporal dementia (MND/FTD)	GGGGCC	1-19	≥24	Intron	chr9:27573528-27573546	V7 only	V7 only
CACNA1A	SCA6	Spinocerebellar ataxia 6 (SCA6)	CAG	4-18	≥21	Coding	chr19:13207858-13207897	V6 & V7	V6 & V7
FGF14	EA	Spinocerebellar ataxia 27B (SCA27B)	GAA	9-249	≥300	Intron	chr13:102161567-102161726	V7 only	V7 only
FMR1	FXS	Fragile X Syndrome (FXS)	GAA	6-40	≥201	Intron	chrX:147912050-147912110	No	V7 only
HTT	HD	Huntington's disease (HD)	CAG	9-26	≥36	Coding	chr4:3074876-3074933	V7 only	V7 only
JPH3	HD-like 2	Huntington disease-like 2 (HDL2)	CTG	6-27	≥41	3′ UTR	chr16:87604287-87604329	V7 only	V6 & V7
NIPA1	ALS	Amyotrophic lateral sclerosis (ALS)	GCG	6-10	≥11	5′ UTR	chr15:22786677-22786701	No	V6 & V7
NOP56	SCA36	Spinocerebellar ataxia 36 (SCA36)	GGCCTG	3-14	≥25	Intron	chr20:2652733-2652757	V6 & V7	V6 & V7
NOTCH2NLC	NIID	Neuronal intranuclear inclusion disease (NIID)	GGC	7-39	≥90	5′ UTR	chr1:149390802-149390829	V7 only	V7 only
PPP2R2B	SCA12	Spinocerebellar ataxia 12 (SCA12)	CAG	7-31	≥51	5′ UTR	chr5:146878728-146878758	V6 & V7	V6 & V7
RFC1	CANVAS	Cerebellar ataxia, neuropathy, and vestibular areflexia syndrome (CANVAS)	AAGGG, ACAGG, AAAAG	11-200 (AAAAG)	≥400 (AAGGG, ACAGG)	Intron	chr4:39348424-39348485	V6 & V7	V7 only
TBP	SCA17	Spinocerebellar ataxia 17 (SCA17)	CAG	25-42	≥43	Coding	chr6:170561907-170562015	V6 & V7	V6 & V7
